# The Chemistry Behind the Folin–Ciocalteu Method for the Estimation of (Poly)phenol Content in Food: Total Phenolic Intake in a Mediterranean Dietary Pattern

**DOI:** 10.1021/acs.jafc.3c04022

**Published:** 2023-11-10

**Authors:** Maria Pérez, Inés Dominguez-López, Rosa M. Lamuela-Raventós

**Affiliations:** † Polyphenol Research Group, Department of Nutrition, Food Science and Gastronomy, XIA, Faculty of Pharmacy and Food Sciences, Institute of Nutrition and Food Safety (INSA-UB), University of Barcelona, 08028 Barcelona, Spain; § Consorcio CIBER, M.P. Fisiopatología de la Obesidad y Nutrición (CIBERObn), Instituto de Salud Carlos III (ISCIII), 28029 Madrid, Spain

**Keywords:** antioxidant, total phenolic content, bioactive compounds, structure−activity relationship, virgin olive oil, wine

## Abstract

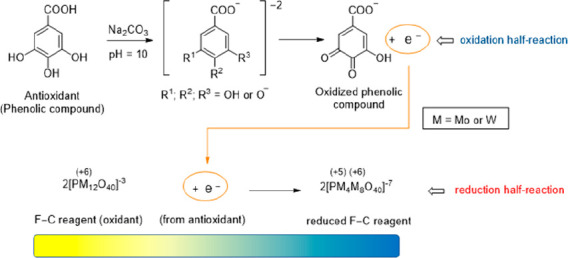

The Folin–Ciocalteu assay is a reference method for the quantification of total (poly)phenols in food. This review explains the fundamental mechanism of the redox reaction on which the method is based and looks at some of the practical considerations concerning its application. To accurately estimate the antioxidant capacity of (poly)phenolic compounds, a thorough knowledge of their structural characteristics is essential, as the two are closely associated. Therefore, to help researchers interpret the results of the Folin–Ciocalteu method, this review also summarizes some of the main phenolic structural features. Finally, we have used the Folin–Ciocalteu method to estimate the total phenolic intake associated with high adherence to a Mediterranean diet, ranked as one of the healthiest dietary patterns, which is characterized by a high consumption of (poly)phenol-rich food such as wine, virgin olive oil, fruits, vegetables, whole grains, nuts, and legumes.

## 1 Introduction

The Mediterranean diet is characterized by high consumption of fruits, vegetables, whole grains, legumes, and olive oil, moderate intake of wine, fish, and poultry, and low consumption of red meat and dairy products. Ranked as the healthiest diet in the world by the U.S. News and World Report,^1^ it also has an added value of sustainability, being typically based on locally produced, traditional, and seasonal foods.^2^ The health benefits of the Mediterranean diet are partly attributed to the effects of (poly)phenols,^3^ the daily intake being around 800–900 mg. Apart from coffee, a principal source of dietary phenols, the diet of Mediterranean countries is distinguished from the dietary habits of northern Europe by the consumption of wine, olives, and virgin olive oil, all rich in (poly)phenols.^4^

In the field of phenolic analysis, the Folin–Ciocalteu (F–C) assay was initially applied to study wine, but it has since become the reference method to determine and quantify phenolic compounds in a wide variety of foods and biological samples due to its simplicity and reproducibility.^5,6^ Despite its popularity, the F–C test is not specifically designed for phenolic compounds, as the reagent could be reduced by other nonphenolic compounds also present in the sample, with the risk of content overestimation.^7,8^ Numerous methods exist to gauge the overall phenolic content and antioxidative potential of fruits and vegetables, relying on chemical reactions that encompass the transfer of hydrogen atoms (HATs) or single electrons (SETs). For instance, the oxygen radical absorbance capacity (ORAC) test is HAT based, while the F–C and ferric reducing antioxidant power (FRAP) assays involve SET reactions. On the other hand, Trolox equivalent antioxidant capacity (TEAC) assays incorporate elements of both SET and HAT mechanisms. It should be noted that the values obtained from these various measurements often diverge, especially when comparing the results of SET and HAT assays. The disparities can be attributed to several factors: the underlying mechanisms, the use of different reference standards (such as gallic acid, Trolox, quercetin, etc.) to express antioxidant activity, the varying sensitivities of compounds to each test, and the complex nature of food matrices, which frequently cause interferences and matrix effects.^9^ These discrepancies have been untangled in a recent publication combining data from various food indexes and electrochemical studies in a global approach.^10^ In another comparative study, the antioxidant capacity of plant extracts measured by various methods was linked to the concentration of phenolic compounds as determined by the F–C technique. The results of the DPPH and ABTS assays were found to be strongly correlated with those of the F–C method (*R* = 0.939 and 0.966, respectively). Similarly, a robust correlation was observed between the ferric-reducing potential, as determined by the FRAP assay, and the total phenolic content (*R* = 0.906).^11^ The selection of the F–C assay over other techniques is usually based on its reputation for reliability, having a long history of use and acceptance in the scientific community. Moreover, it is relatively cost effective compared to other methods, rendering it accessible for researchers with limited budgets. As the F–C assay is sensitive and can quantify a wide range of phenolic compounds, it is suitable for analyzing complex phenolic mixtures found in fruits, vegetables, and other foods. Additionally, it can be easily integrated into various laboratory setups and is compatible with common laboratory equipment.

The oxidizing F–C reagent reacts with reducing agents (antioxidants) to form a soluble, vividly blue complex, although its structure and the mechanism of its reactivity with phenolic compounds have not been fully determined.^12^ The diverse class of chemical compounds known as (poly)phenols, which are among the most significant plant antioxidants, is distinguished by the presence of a phenol functional group, which consists of a hydroxyl (−OH) group directly attached to an aromatic ring. The study of the structure–activity relationships of the main dietary phenolics in the F–C reaction has found that the antioxidant activity of phenolic compounds is strongly affected by their structural features.

The aim of this review is to summarize the chemistry behind the F–C assay, focusing on the reagent itself, the redox reaction that takes place during the assay, as well as the relationship between the structural elements of the main dietary phenolic compounds and their antioxidant capacity. Moreover, the total phenolic intake associated with high adherence to a Mediterranean diet^13^ quantified by F–C analysis is assessed.

## 2 Folin–Ciocalteu Reagent

**Figure 1:**
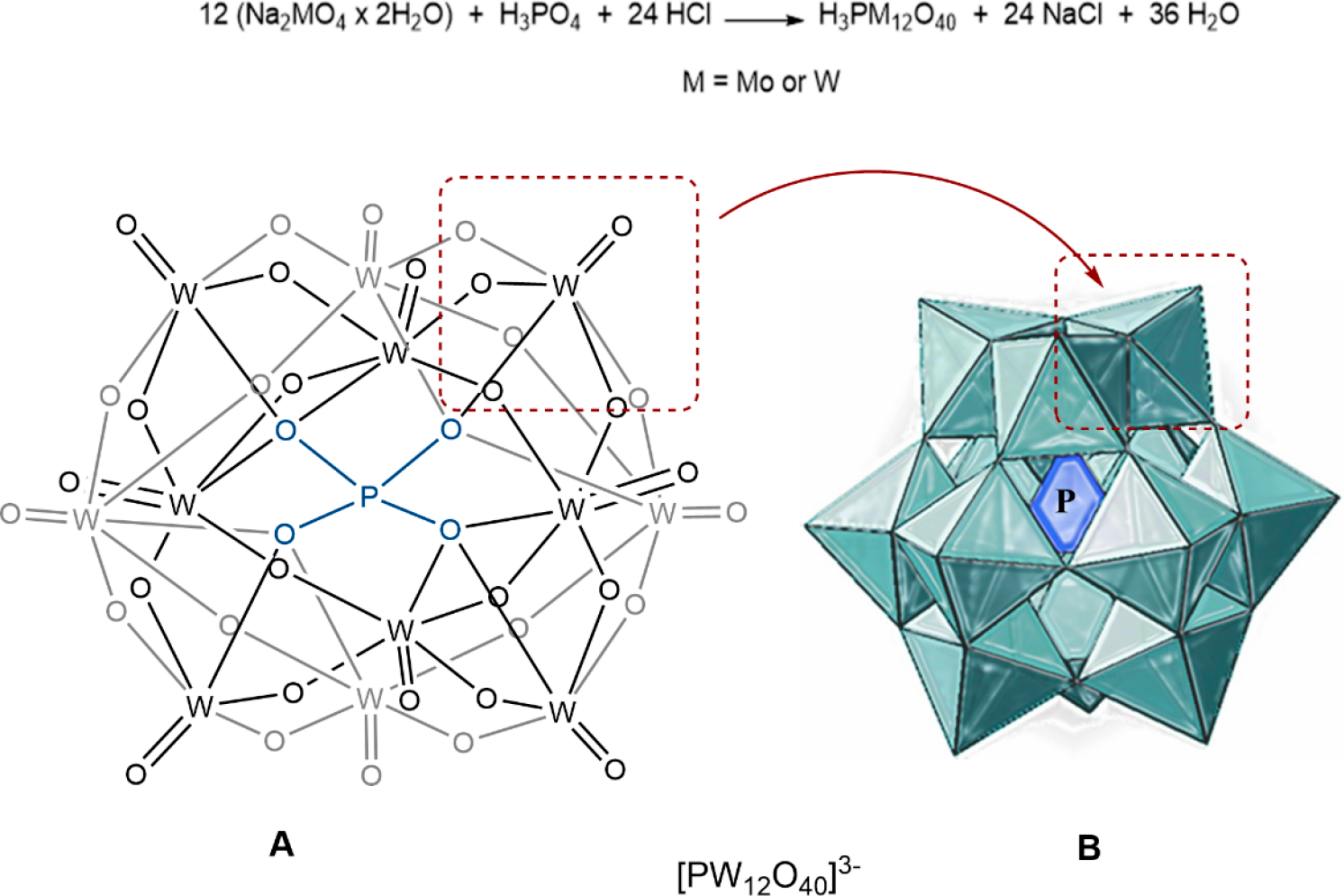
Preparation of the Folin–Ciocalteu reagent. α-Keggin structure of the anionic derivative [PW_12_O_40_]^3–^ (A). Polyhedral model form (B).^19^

Although the F–C reagent is readily available on the market, it can also be prepared following the original protocol^14^ by boiling a mixture made of sodium tungstate (Na_2_WO_4_·2H_2_O, 100 g), sodium molybdate (Na_2_MoO_4_·2H_2_O, 25 g), concentrated hydrochloric acid (100 mL), 85% phosphoric acid (50 mL), and water (700 mL) for 10 h (Figure 1). The process generates a yellow solution composed of the complex compounds, phosphomolybdic acid (H_3_PMo_12_O_40_) and phosphotungstic acid (H_3_PW_12_O_40_). Lithium sulfate (150 g, Li_2_SO_4_·4H_2_O) is added after boiling to reduce the formation of precipitates. If the reagent turns green because of contaminating reductants, its quality can be restored by adding a few drops of bromine or a small amount of 30% hydrogen peroxide.^15^

The precise chemical structure of the F–C reagent is unknown; however, it is described as a complex mixture of phosphotungstic and phosphomolybdic acids that is reduced throughout the assay to produce a blue chromophore with a maximum absorbance at 765 nm.^14−16^ In 1933, Keggin solved the structure of the acid H_3_PW_12_O_40_ using powder X-ray diffraction (see Figure 1).^17^ The α-Keggin structure of the anionic derivatives of phosphotungstic and phosphomolybdic acids has the general formula [XM_12_O_40_]^*n*−^,^18^ where X is the heteroatom (in the F–C reagent, X is pentavalent phosphorus P(V)), M is the addendum atom (molybdenum, Mo, and/or tungsten, W), and O is oxygen. The structure has tetrahedral symmetry and is comprised of one phosphorus surrounded by four oxygen atoms (depicted in blue in Figure 1). The 12 octahedral MO_6_ units that surround the core heteroatom are connected by the nearby oxygen atoms.

## 3 Redox Reaction in the Folin–Ciocalteu Assay

**Figure 2:**
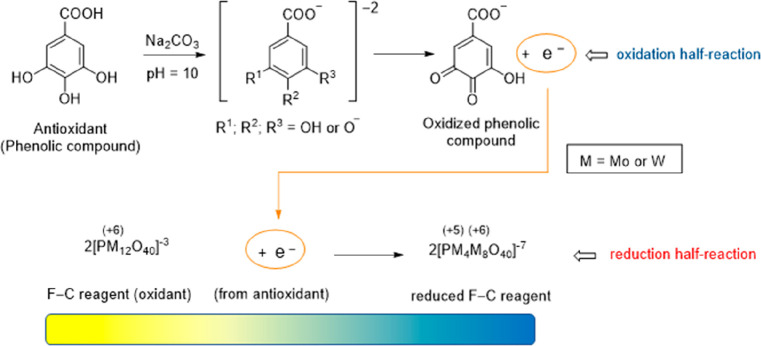
General redox reaction in the Folin–Ciocalteu assay. Metal complex species according to Munteanu.^7^

The F–C method is based on an electron-transfer reaction in which the antioxidant species acts as the electron donor and the F–C reagent acts as the oxidant (see Figure 2).

The reduction of the anionic derivatives of phosphotungstic and phosphomolybdic acids by antioxidants causes a color shift from yellow to blue, and the magnitude of the color shift when the reaction is complete is directly proportional to the reducing activity of the phenolic compounds. The reducing capacity of an antioxidant is frequently measured as gallic acid equivalents (GAE).^8^ In more detail, the transfer of electrons from phenolic compounds to phosphomolybdic/phosphotungstic acid complexes in an alkaline solution creates blue complexes that are detected spectroscopically at about 760 nm. (Poly)phenols react with the F–C reagent only under basic conditions (pH of 10, adjusted by a sodium carbonate solution). Although the exact chemical composition of the F–C reaction is unknown, a series of reversible one- or two-electron reactions promoted by the phenolic compounds at basic pH change the initial yellow F–C reagent (H_3_PMo_12_O_40_ + H_3_PW_12_O_40_) to blue species, which may be (PMoW_11_O_40_),^4−8^ (PM_12_O_40_)^7–^ (M = Mo or W),^7^ or Mo_8_O_23_ + W_8_O_23_.^20^ It is assumed that molybdates are more easily reduced than tungstates in heteropoly salts; hence, the electron-transfer reaction takes place between the phenolic compound and Mo(VI), and some of the Mo^6+^ in the complex are reduced to Mo^5+^ by accepting an electron from the phenolic antioxidant (Figure 2).

## 4 Practical Considerations Regarding the Folin–Ciocalteu Assay

The F–C assay is a useful method for determining the antioxidant activity of phenolic compounds as it is easy to use, consistent, and reliable. Nonetheless, the reaction conditions should be chosen carefully as the accuracy of the test is influenced by factors such as pH, temperature, and reaction duration. As interference issues in the F–C assay strongly depend on the food matrix and the variable reducing capacity of nonphenolic compounds, there are no simple guidelines. Nevertheless, some authors have studied the use of different methods to clean up the interference substances and alternative F–C reacting conditions to limit TPC overestimation.^21,22^

### 4.1 Standard for Calibration

Tannic acid has long been used as a reference for calibration curves when determining the total phenolic content (TPC) of wines.^5^ However, because the content of tannic acid can differ among wines and spirits, Singleton et al.^5^ substituted it for GAE as a reference for reporting F–C results. The gallic acid added to the wine was quantitatively recovered, and a mixture of natural phenolic compounds of various classes produced an absorbance equal to the total of their individual contributions, indicating that chemical deviances from Beer’s rule were largely absent in the F–C system. Depending on the thickness of the optical cuvette, the minimum limit of quantification is 3 mg GAE/L. Although gallic acid is now routinely used as a standard for calibration curves, equivalents of catechin,^23,24^ tannic acid,^25^ chlorogenic acid,^26^ caffeic acid,^27^ and ferulic acid^28^ have also occasionally been used, requiring standardization of the reported results.^29^ Caffeic acid,^30^ gallic acid,^31^ and hydroxytyrosol (HTyr)^32^ calibration curves were utilized to measure phenolic compounds in virgin olive oil extracts. Twelve different extra virgin olive oils were analyzed using various methods, and their phenolic content was statistically compared using two-tailed paired *t* tests. Results from the F–C assay (expressed as HTyr/20 g of oil) before and after acid hydrolysis were statistically similar to acid hydrolysis–HPLC results (HTyr + tyrosol).^32^

### 4.2 pH in the Folin–Ciocalteu Assay

Phenolic compounds only react with the F–C reagent in basic conditions. A sodium carbonate solution is added to the mixture containing the sample and the acidic F–C reagent to bring the pH level to approximately 10, avoiding excessive alkalinity. Sodium hydroxide and sodium cyanide have also been successfully used for this purpose.^5^ A comparable approach based on the generation of phosphomolybdenum blue using a reagent without tungstate was described for the evaluation of antioxidant capacity in an acidic medium at a high temperature,^33^ but this alternative method has not been tested with a wide range of antioxidants.^34^

### 4.3 Temperature and Time of Sample Incubation

In the F–C assay, the sample must be incubated with the reagent for 1 h, after which the absorbance is measured at 760 nm at room temperature. The blue color is quite stable at room temperature, so measurement of the standard, blank, and sample set at 760 nm after 6 h gives similar results to those after 1 h, albeit the standard deviation is higher.^5^ The color may emerge more rapidly at a warmer temperature, but higher temperatures (>40 °C) cause the color to disappear more quickly.

### 4.4 Solvent Used in the Folin–Ciocalteu Assay

The conventional F–C reagent can only be used with water-soluble antioxidants,^29^ and the reaction media is treated with lithium sulfate to prevent the precipitation of sodium complexes.^14^ Thus, for the simultaneous analysis of lipophilic and hydrophilic antioxidants, the F–C method was modified and standardized using an isobutanol and water medium with sodium hydroxide.^35^ Although this alternative procedure is not routinely applied, it has been successfully used to test hydrosoluble compounds such as ascorbic, gallic, caffeic, ferulic, and rosmarinic acids, Trolox, quercetin, catechin, glutathione, and cysteine as well as lipophilic antioxidants like butylated hydroxyanisole, butylated hydroxytoluene, *tert*-butylhydroquinone, lauryl gallate, and β-carotene. There is a need for further studies to evaluate the F–C method with other lipophilic antioxidants.

## 5 Structure–Activity Relationships of (Poly)phenols in the Folin–Ciocalteu Assay

To ascertain the impact of the highly variable phenolic structures on the results of the F–C assay, in this section, we explore how the structural properties of the major dietary phenolic compounds are related with their reducing capacity and, consequently, their antioxidant ability. Several studies have employed the F–C method to assess the antioxidant activity of samples containing a broad range of structurally diverse phenolic compounds, whereas more in-depth research on phenolic structure–activity relationships has focused mainly on phenolic acids and flavonoids.

### 5.1 Phenolic Acids

**Figure 3:**
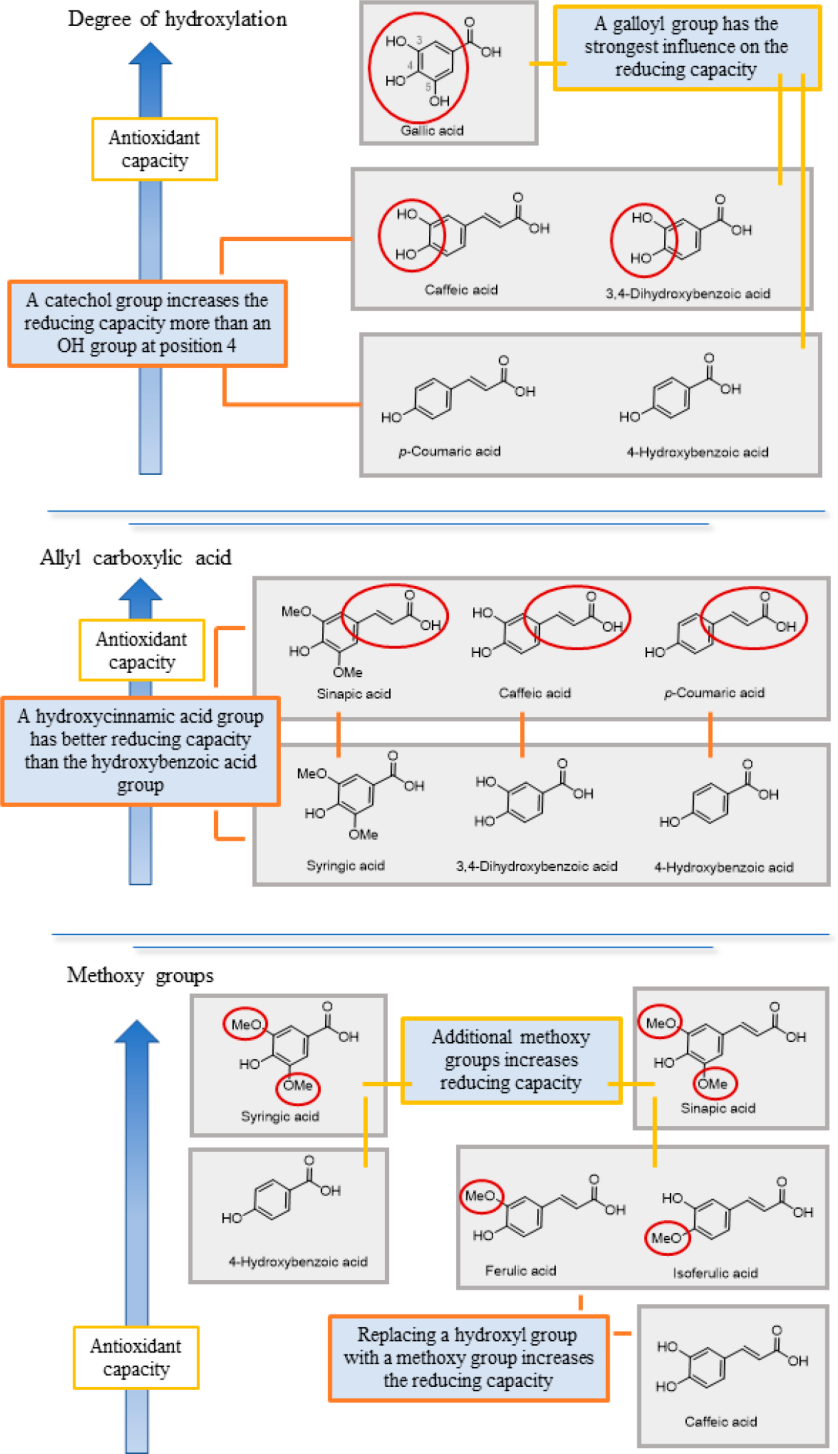
Key factors in the reducing capacity of phenolic acid derivatives: degree of hydroxylation, allyl carboxylic acid, and methoxy groups.

The ability of phenolic acids to scavenge free radicals depends on the quantity and position of the hydroxyl and methoxy groups in their molecules (Figure 3).^36,37^ The galloyl group has the most positive effect on phenolic reducing capacity, which explains why gallic acid, a 3,4,5-trihydroxybenzoic acid, is the strongest antioxidant in the phenolic acid group. Additionally, compounds with a catechol group rather than a single hydroxyl group at position 4 have higher reducing capacities, which is the case of caffeic acid in comparison with *p*-coumaric acid and 3,4-dihydroxybenzoic acid in comparison with 4-hydroxybenzoic acid.^10,38^ A likely explanation is that the stabilization of the phenoxyl radical by an intramolecular hydrogen bond enhances antioxidant activity.^39^

Despite having the same number of hydroxyl and methoxy groups in the same position, hydroxycinnamic acids have a stronger reducing capacity than hydroxybenzoic acids, probably because the former have higher resonance stabilization.^37,38^ Hence, a higher reducing capacity is found for sinapic acid versus syringic acid, caffeic acid versus 3,4-dihydroxybenzoic acid, and *p*-coumaric acid versus 4-hydroxybenzoic acid.

Additionally, the response of phenolic compounds in the F–C assay is improved if they bear a methoxy group instead of hydrogen atoms at the corresponding positions. This accounts for the slightly higher values obtained for syringic acid compared to 4-hydroxybenzoic acid and for sinapic acid compared to ferulic and isoferulic acids.^37,38^ Furthermore, in hydroxycinnamic acids, replacing a hydroxyl group with a methoxy group (an electron donor) can improve the radical scavenging activity and boost the reducing capacity, explaining why ferulic acid has more reducing power than caffeic acid.^36,37,39,40^

### 5.2 Flavonoids

**Figure 4:**
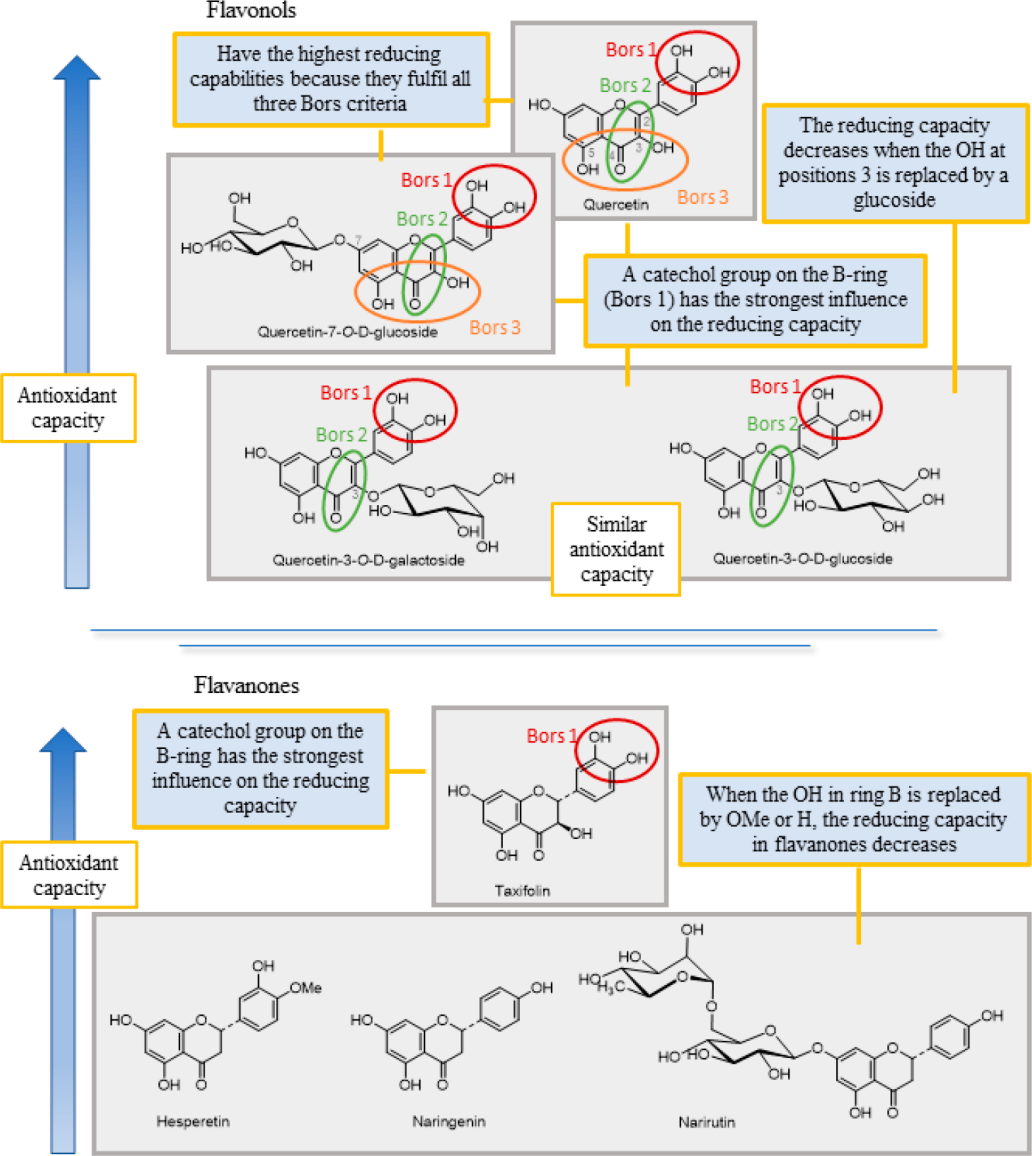
Key factors in the reducing capacity of flavonols and flavanones.

Three structural properties, based on Bors criteria, have been postulated to explain the antioxidant capacity of flavonoids^41^ (Figure 4). The presence of a catechol group on the B ring (Bors 1) increases the stability of the resulting antioxidant radical; a 2,3 double bond conjugated to a 4-oxo group on the C ring (Bors 2) allows electron delocalization; the presence of OH groups at positions 3 and 5 in combination with a 4-oxo group facilitates electron delocalization via hydrogen bonds (Bors 3). Previous studies have found that the number and placement of OH groups in flavonoids, particularly those on the B ring, and glycosylation affect the F–C assay results.^10,37,42^ The flavonoids without a hydroxyl group (e.g., *trans*-chalcone, flavone, and isoflavone) have no radical scavenging capacity.^36^ As expected, flavonols and flavanols have stronger reducing capabilities than other flavonoids, followed by some flavanones. Of the three Bors criteria, Bors 1 is thought to have the greatest influence on the reducing capacity, because flavanols only fulfill Bors 1 despite having equivalent reducing power to the flavonols.^38^

In the flavonol subgroup, the catechol group on the B ring (fulfilling Bors 1) has the greatest influence on the flavonol reducing capacity, which explains the high values found for quercetin, quercetin-7-*O*-d-glucoside, quercetin-3-*O*-d-galactoside, and quercetin-3-*O*-d-glucoside (Figure 4). As it meets all three Bors criteria, quercetin is the most powerful reducing agent, followed by quercetin-7-*O*-glucoside. The reducing capacity is lower in quercetin-3-*O*-d-glucoside and quercetin-3-*O*-d-galactoside because the OH at position 3 is replaced by a sugar residue, a weaker electron-donating group than OH. However, as their values were not significantly different, it is assumed that the type of sugar residue does not influence the reducing capacity.^38^ In flavanones, the presence of a catechol group (Bors 1) has the strongest effect on their reducing abilities, which explains why taxifolin outperforms hesperetin, narirutin, and naringenin.^38^

**Figure 5:**
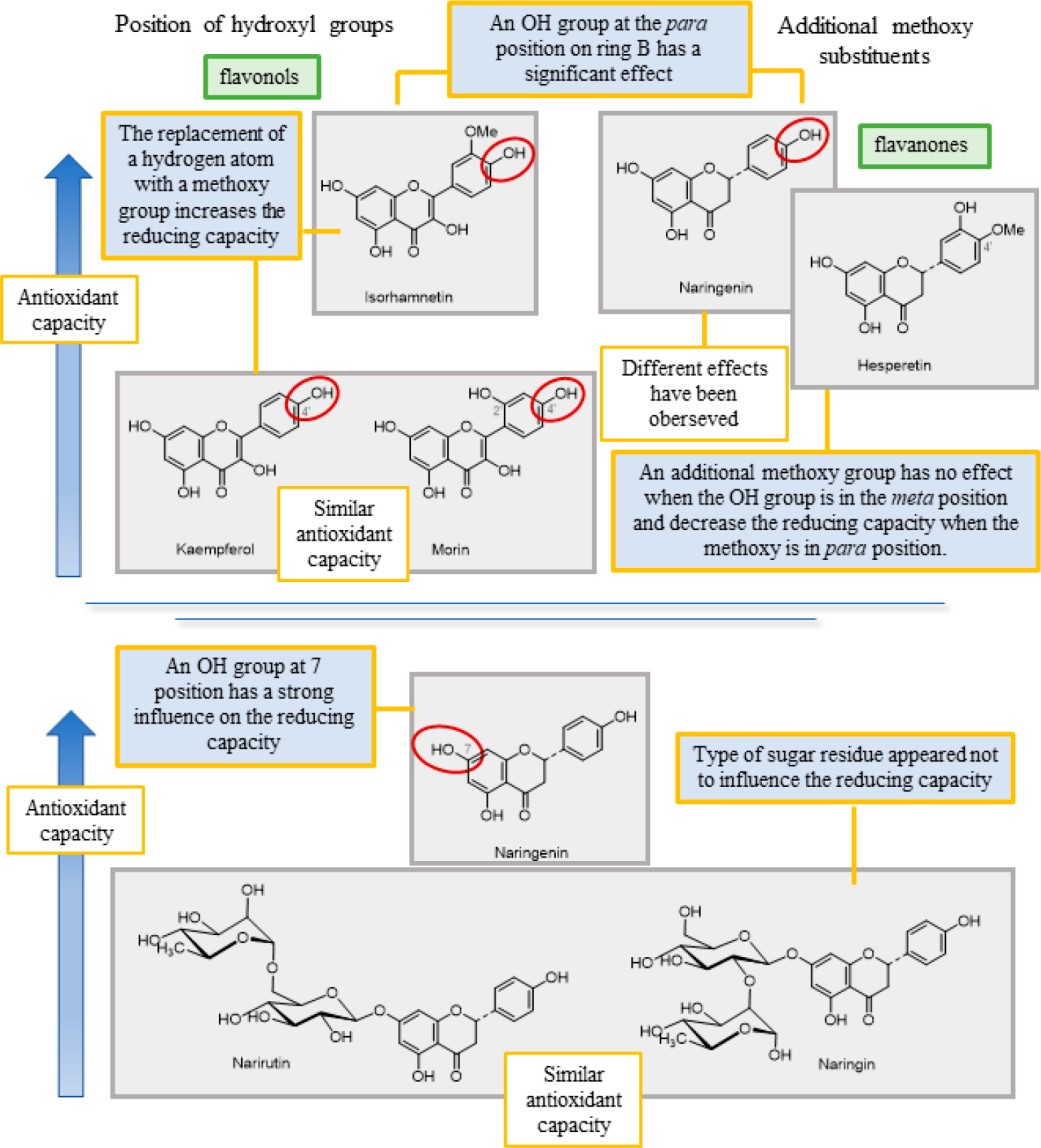
Key factors in the reducing capacity of flavonoids: positions of the hydroxyl groups and the presence of additional methoxy substituents.

Generally, OH groups in the ortho and para positions appear to confer greater reducing capacity than those in the meta position due to the stabilization of the phenoxyl radical by intramolecular hydrogen bonds (Figure 5).^37,39,43^ However, in flavonols, the presence of a hydroxyl group at position 4′ was found to have a substantial effect whereas a hydroxyl group at position 2′ had a minimal effect, explaining why there was no significant difference in the reducing capacity between morin and kaempferol. Replacing a hydrogen atom with a methoxy group increases the reducing ability, hence the higher value of isorhamnetin compared to kaempferol,^38^ whereas replacing a hydroxyl group with a methoxy group has the reverse effect (Cai et al., 2006;^36^ Ma and Cheung, 2007;^37^ Shahidi et al., 1992^39^). Despite having an extra methoxy group on the B ring, according to Platzer, the reducing ability of hesperetin does not differ significantly from that of naringenin because the hydroxyl group is in the meta position.^38^ However, Ma et al. reported that the presence of a methoxy group instead of a hydroxyl group at position 4′ decreases the reducing power of hesperetin compared to naringenin as the resulting methoxy-substituted phenoxy radical cannot be stabilized by intramolecular hydrogen bonding.^37^

The presence of a hydroxyl group at position 7 also has a substantial impact, hence the noticeably greater reducing ability of naringenin compared to narirutin.^44^ As in flavonols, the reducing capacity of flavanones appears to be unaffected by the type of sugar residue, which explains the identical values found for narirutin and naringin.^38^

In summary, the structure–activity relationship of phenolic antioxidants in the F–C assay has been explored in phenolic acids, flavonols, and flavanones but not flavanols. The antioxidant activity of primary dietary phenolic compounds can be predicted based on their structural properties. While the F–C assay results are mostly explained by how many Bors criteria are met, the degree of hydroxylation and the locations of the hydroxyl and methoxy groups are key variables in the reducing capacity when none of the Bors principles are applicable.

## 6 Total Phenolic Intake with High Adherence to a Mediterranean Diet

A high adherence to a Mediterranean diet is associated with more beneficial health outcomes compared to a low adherence due to a higher intake of total (poly)phenols as well as specific phenolic compounds such as flavonoids, anthocyanins, and lignans.^45^Table 1 lists the main food sources of (poly)phenols and the amount of (poly)phenols consumed when following a Mediterranean diet according to the validated MEDAS (14-point Mediterranean Diet Adherence Screener) questionnaire.^46^ The TPC data were obtained from Polyphenol Explorer Database, which is based on average values from published studies.^47,48^ However, to obtain the TPC of foods that are typically consumed after cooking, the original literature sources were examined.

**Table 1:**
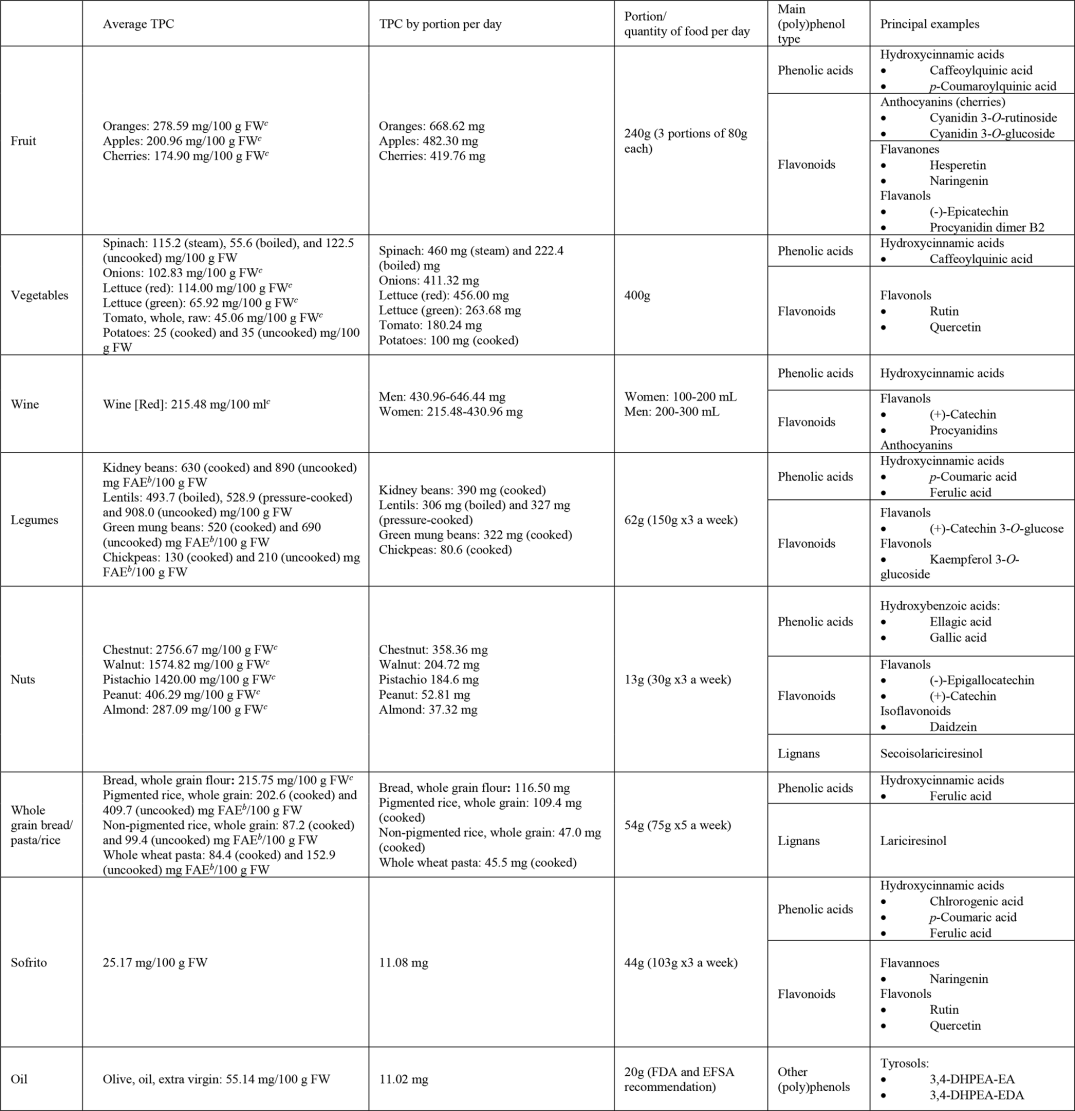
Average Total Phenolic Content (TPC), TPC by Portion, and the Main (Poly)phenols in Foods Consumed in the Mediterranean Diet^a^

a Portions were defined according to the recommendations of the Mediterranean diet^46^ and in raw food; the TPC data were obtained from the Phenol Explorer Database.^48,58^

b mg/100 g FW using equivalents of ferulic acid.

c Polyphenol Explorer Database overall data.

According to the MEDAS questionnaire, fruits constitute one of the main sources of (poly)phenols for the Mediterranean population, with oranges being the fruit consumed with the highest TPC, followed by apples and cherries.^46^ Considering that three portions of fruit per day are recommended in the Mediterranean dietary pattern, which is equivalent to 240 g, these foods provide 419.76–668.62 mg of (poly)phenols per day. Phenolic acids are commonly found in fruits, the most significant being hydroxycinnamic acids such as caffeoylquinic acid and *p*-coumaroylquinic acid. Fruits also contain three types of flavonoid compounds: flavanones (e.g., hesperetin and naringenin), flavanols (e.g., (−)-epicatechin and procyanidin dimer B2), and anthocyanins (e.g., cyanidin 3-*O*-rutinoside and cyanidin 3-*O*-glucoside in cherries). Anthocyanins are responsible for the red, purple, and blue colors of many fruits.

The TPC in vegetables is highly variable depending on the vegetable and if they are fresh or cooked. Among the most frequently consumed, those with the highest content of (poly)phenols measured with the F–C assay are spinach, onions, red and green lettuce, and, finally, tomatoes and potatoes. Spinach is commonly consumed raw in salads or boiled/steamed, and its TPC varies depending on the preparation method.^49^ In contrast, potatoes are usually boiled, resulting in a reduction of TPC from 35 to 25 mg/100 g FW.^50^ Following the recommended consumption of 400 g of vegetables per day, the average daily total phenolic intake from this source would range from 100 to almost 460 mg. The main phenolic compounds present in these vegetables are phenolic acids such as caffeoylquinic acids (chlorogenic acid) and flavonols such as kaempferol and quercetin.

The Mediterranean diet is characterized by a moderate consumption of wine, mainly red, with meals. The TPC in red wine depends on the type of grapes used and the wine making process, among other factors, but on average, it is 215.48 mg/100 mL. In the Mediterranean dietary pattern,^2^ red wine represents one of the main sources of phenolic compounds, providing a daily intake of 215.48–430.96 mg for women and 430.96–646.44 mg for men. The main (poly)phenols in red wine are hydroxycinnamic acids and flavonoids such as anthocyanins and flavanols ((+)-catechin and procyanidins).

Legumes, particularly beans, are recognized as a very good source of (poly)phenols, although there is a notable difference between cooked and uncooked legumes. In uncooked kidney beans, the TPC can reach up to 870 mg per 100 g compared to about 630 mg per 100 g after boiling. Consuming 62 g (150 g × 3 a week) of kidney beans provides 390 mg of phenolic compounds.^51^ In second place are lentils, which have the highest TPC among legumes when analyzed in raw form (908 mg/100 g fresh weight (FW)), the amount also decreasing drastically after cooking, with boiled lentils containing 493.7 mg/100 g FW and pressure-cooked lentils 529 mg/100 g FW.^52^ Green mung beans are another valuable source of (poly)phenols, a portion of 62 g providing 322 mg of phenolic compounds when cooked. The TPC of chickpeas is slightly lower, decreasing by 35.6% after cooking.^51^ According to the guidelines of the Mediterranean diet, legumes, particularly kidney beans, lentils, and green mung beans, constitute one of the primary sources of total phenolic intake, despite their reduction by cooking. (+)-Catechin 3-*O*-glucose and kaempferol 3-*O*-glucoside are flavanols found in legumes, which are also a good source of phenolic acids such as hydroxycinnamic acids (*p*-coumaric and ferulic acids).

Among nuts, the highest TPC is found in chestnuts, followed closely by walnuts and pistachios, with a lower content in peanuts and almonds. The main (poly)phenols in nuts are hydroxybenzoic acids (e.g., ellagic acid and gallic acid), lignans (e.g., secoisolariciresinol), flavonoids (e.g., flavanols such as (−)-epigallocatechin and (+)-catechin), and isoflavonoids (e.g., daidzein).

In the Mediterranean diet, there is a preference for whole grain foods, which have a higher content of bioactive compounds such as fiber and (poly)phenols, over refined foods. Bread, rice, and pasta made with whole grains are good sources of lignans (lariciresinol) and hydroxycinnamic acids such as ferulic acid. Cooking was found to reduce the average TPC in pigmented rice by about 50% (from 410 to 203 mg ferulic acid equivalents (FAE)/100 g FW) but had no significant effect on the average TPC of nonpigmented rice, which remained quite constant (87.2 mg FAE/100 g FW).^53^ A 54 g portion of whole wheat pasta, despite the reduction of TPC after cooking (57.7%), provides 45.5 mg of (poly)phenols per day.^54^

Sofrito, a traditional sauce in Mediterranean cuisine prepared by sautéing onions, garlic, and tomatoes in olive oil, is reported to contain 25.17 mg of phenolic compounds per 100 g of FW.^45^ Among these compounds are various types of phenolic acids, including hydroxycinnamic acids such as chlorogenic acid, *p*-coumaric acid, and ferulic acid.

Extra virgin olive oil (EVOO) is the main source of fat in the Mediterranean diet. The recommended daily intake of EVOO, according to the Food and Drug Administration (FDA) and European Food Safety Authority (EFSA), is 20 g per day, which would provide approximately 11.02 mg of (poly)phenols.^55^ The specific types of (poly)phenols found in EVOO are tyrosols and secoiridoids such as 3,4-DHPEA-EA and 3,4-DHPEA-EDA. It is worth noting that the TPC in EVOO varies according to factors such as the olive variety and stage of ripeness, the production process, and storage conditions.^56,57^

## 7 Conclusions

Although the precise chemical composition of the F–C reagent is unknown, the F–C assay is based on the reduction of a yellow phosphotungstate–phosphomolybdate complex by antioxidants (reductants) to a blue chromogen. The reducing capacities of the major dietary phenolic compounds can be predicted based on their structural features. As the structure of phenolic compounds conditions their antioxidant power, the results of the F–C assay will depend on the content of individual (poly)phenols in the sample.

The F–C assay has been widely used in studies to measure the TPC in foods or extracts and is regarded as a reference method in this regard. The Mediterranean diet is characterized by the consumption of many (poly)phenol-rich foods, such as fruits, vegetables, legumes, wine, and nuts, which could be partly responsible for its demonstrated health benefits. However, it is important to note that the F–C assay measures the TPC, and not all phenolic compounds have the same bioactivity or health impact. Therefore, more research is needed to understand the health effects of specific phenolic compounds in the Mediterranean diet.

## Abbreviations Used

EVOO: extra virgin olive oil
FAE: ferulic acid equivalents
F–C: Folin–Ciocalteu
FW: fresh weight
GAE: gallic acid equivalents
TPC: total phenolic content
